# National religiosity eases the psychological burden of poverty

**DOI:** 10.1073/pnas.2103913118

**Published:** 2021-09-20

**Authors:** Jana B. Berkessel, Jochen E. Gebauer, Mohsen Joshanloo, Wiebke Bleidorn, Peter J. Rentfrow, Jeff Potter, Samuel D. Gosling

**Affiliations:** ^a^Mannheim Centre for European Social Research, University of Mannheim, 68131 Mannheim, Germany;; ^b^Institute of Psychology, University of Copenhagen, 1165 Copenhagen, Denmark;; ^c^Department of Psychology, Keimyung University, Daegu 42601, Republic of Korea;; ^d^Department of Psychology, University of Zurich, 8006 Zurich, Switzerland;; ^e^Department of Psychology, University of Cambridge, Cambridge CB2 1TN, United Kingdom;; ^f^Atof Inc., Cambridge, MA 02139;; ^g^Department of Psychology, University of Texas at Austin, Austin, TX 78712;; ^h^Melbourne School of Psychological Sciences, University of Melbourne, Parkville VIC 3010, Australia

**Keywords:** socioeconomic status, well-being, religiosity, economic development

## Abstract

Lower socioeconomic status (SES) harms psychological well-being, an effect responsible for widespread human suffering. This effect has long been assumed to weaken as nations develop economically. Recent evidence, however, has contradicted this fundamental assumption, finding instead that the psychological burden of lower SES is even greater in developed nations than in developing ones. That evidence has elicited consternation because it suggests that economic development is no cure for the psychological burden of lower SES. So, why is that burden greatest in developed nations? Here, we test whether national religiosity can explain this puzzle. National religiosity is particularly low in developed nations. Consequently, developed nations lack religious norms that may ease the burden of lower SES. Drawing on three different data sets of 1,567,204, 1,493,207, and 274,393 people across 156, 85, and 92 nations, we show that low levels of national religiosity can account for the greater burden of lower SES in developed nations. This finding suggests that, as national religiosity continues to decline, lower SES will become increasingly harmful for well-being—a societal change that is socially consequential and demands political attention.

## Significance

According to a fundamental assumption in the social sciences, the burden of lower socioeconomic status (SES) is more severe in developing nations. In contrast to this assumption, recent research has shown that the burden of lower SES is less—not more—severe in developing nations. In three large-scale global data sets, we show that national religiosity can explain this puzzling finding. Developing nations are more religious, and most world religions uphold norms that, in part, function to ease the burden of lower SES and to cast a bad light on higher SES. In times of declining religiosity, this finding is a call to scientists and policymakers to monitor the increasingly harmful effects of lower SES and its far-reaching social consequences.

Lower socioeconomic status (SES) is harmful to psychological well-being (1, 2). According to one influential study, for example, people of lower SES are about four times more likely to suffer negative affect than their higher SES counterparts (3). These harmful effects of lower SES are costly for economies and societies (4, 5), but above all, they pose a major humanitarian problem (6, 7). But does lower SES necessarily diminish well-being or can those harmful effects be buffered? One long-held, optimistic assumption (8, 9) has been that the harmful effects of lower SES will weaken as nations become more economically developed (10, 11). This assumption is based on the idea that developed nations provide more welfare services, thereby allowing people of lower SES to better meet their basic needs (10). Meeting basic needs should, in turn, promote well-being (10, 12).

The fundamental assumption that lower SES is most harmful to well-being in developing nations has its roots in the classic sociological writings of Max Weber (13). Indeed, early empirical evidence supported this assumption (10, 14). Scholars, for example, found a sizable association between lower SES and diminished well-being in developing nations (Bangladesh, the Dominican Republic, and Romania) but a much weaker association in developed nations [Latvia, Singapore, and South Korea (10)].

Recently, however, large-scale, comprehensive data became available for almost the entire planet, and the results revealed a very different pattern. The associations between lower SES and diminished well-being were largest in developed nations, not in developing ones (15–17). This finding was sobering for politicians, social scientists, and the general public alike because they had all put their faith in the economic development of nations. Evidently, though, the economic development of nations is not the long-sought panacea for the psychological burden of lower SES. It even seems as if economic development amplifies that burden. But why?

A few initial explanations have been proposed to answer that question, all of which focus on some economic features of nations (15, 17). For instance, one prominent explanation holds that higher SES is a cherished value in developed nations (17, 18), such that people of lower SES fail to meet this value—a failure that constitutes yet another blow to their well-being (19, 20).

Here, we test a very different explanation: It is not a nation’s economic development per se that alters the psychological burden of lower SES, but a tremendously important covariate of it—national religiosity (21, 22). Eminent thinkers, from Voltaire to Durkheim, have pointed to the role of religion in creating and maintaining norm-abiding groups (23, 24). The resulting social norms hold prominent positions in theories of the emergence and perpetuation of culture (25, 26) and, ultimately, human evolution (27, 28).

Among the religious norms that enable cultural groups to thrive is a set relevant for SES. That set eases the burden of lower SES (“The poor are admitted into Paradise before the rich, by five hundred years;” Vol. 5, Book 37, Hadith 4261, The Qur’an; “For those who are poor and destitute; May I turn into all things they could need;” Ch. 3, Verse 10, Bodhisattvacharyavatara) and it does so, in part, by casting a bad light on higher SES [“It is easier for a camel to go through the eye of a needle than for a rich man to enter the kingdom of God;” Matthew 19:24, The Bible, “The demoniac person thinks: So much wealth do I have today, and I will gain more;” Ch. 16, Verse 13, Bhagavad-Gita (16, 29)]. Consequently, the psychological burden of lower SES should be the lightest in developing nations because those nations are the most religious and, thus, uphold the relevant set of religious norms. Accordingly, the psychological burden of lower SES should be most severe in developed nations because those nations are the least religious and, thus, lack the relevant set of religious norms.

A statistical prerequisite for our hypothesis is that lower SES is more harmful to well-being in nonreligious nations than it is in religious nations (16, 30). Some existing evidence suggests that this prerequisite is met. In one early study of online-daters from 11 European nations, the association between personal income and well-being was stronger in secular than in religious nations [even though all nations were developed (16)]. In another study, the association between severe economic hardship (e.g., shortage of food, medicine, or medical treatment) and well-being was amplified in nonreligious compared to religious nations (31).

In all, we test whether national religiosity accounts for the otherwise puzzling evidence that the association between lower SES and diminished well-being is stronger in developed nations and weaker in developing ones. To bolster the robustness of our conclusions, we relied on three worldwide data sets: the Gallup World Poll [GWP; 1,567,204 people across 156 nations (32)], the Gosling-Potter Internet Personality Project [IPP; 1,493,207 people across 85 nations (33)], and the World Values Survey [WVS; 274,393 people across 92 nations (34); for descriptive statistics, see *SI Appendix*, Table S1]. Additionally, we conducted 15 robustness checks to ensure that our results hold up against a variety of alternative explanations.

## Results

First, we sought to replicate the heretofore puzzling finding that the harmful effects of lower SES on well-being are amplified in developed nations. To this end, we estimated a mixed-effects model with well-being as the criterion and SES (level 1), national economic development (level 2), and their cross-level interaction as the predictors (model 1). The model revealed the expected main effect of SES on well-being: GWP: β = 0.28, 95% CI: 0.27 to 0.29; IPP: β = 0.12, 95% CI: 0.11 to 0.13; WVS: β = 0.20, 95% CI: 0.18 to 0.22. Moreover, the model also revealed that higher national economic development amplified this main effect in two of the three data sets: GWP: β = 0.04, 95% CI: 0.03 to 0.05; IPP: β = 0.03, 95% CI: 0.02 to 0.04; WVS: β = −0.02, 95% CI: −0.04 to 0.0001. As an illustration, in the IPP, the psychological burden of lower SES was comparatively large in developed Norway, *r* = 0.23, 95% CI: 0.21 to 0.24, but that burden was nonsignificant in developing Jamaica, *r* = 0.02, 95% CI: −0.06 to 0.10. The finding that the WVS differed from the other two data sets has relevant implications, on which we elaborate in the *Discussion*.

**Fig. 1.**
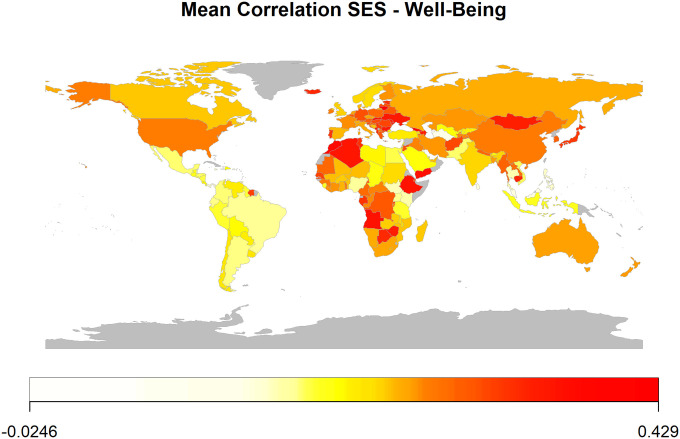
Heatmap of mean zero-order correlations between SES and psychological well-being. For each nation, Pearson correlations were calculated between SES and well-being. Those Pearson correlations were Fisher’s z-transformed, averaged across the three data sets, and back-transformed to correlation coefficients (*SI Appendix*, Table S1 lists the countries in each data set). Lighter colors represent smaller correlations, darker colors larger correlations within each nation. Please note that nations vary in the number of data sets contributing to the averaged correlation coefficients presented in this figure.

Second, we sought to provide a worldwide test of whether the harmful effects of lower SES on well-being are attenuated in more religious nations. Thus, we conducted a mixed-effects model with well-being as the criterion and SES (level 1), national religiosity (level 2), and their cross-level interaction as the predictors (model 2). In this model, too, we found the expected main effect of SES on well-being: GWP: β = 0.28, 95% CI: 0.27 to 0.29; IPP: β = 0.12, 95% CI: 0.11 to 0.12; WVS: β = 0.20, 95% CI: 0.18 to 0.22. Of particular relevance, the model also revealed that national religiosity attenuated this main effect in all three data sets (but note the 90% CI in the case of the WVS): GWP: β = −0.04, 95% CI: −0.05 to −0.03; IPP: β = −0.04, 95% CI: −0.05 to −0.03; WVS: β = −0.02, 90% CI: −0.04 to −0.0004. Fig. 1 depicts the zero-order correlations between SES and well-being across the entire world.

**Fig. 2.**
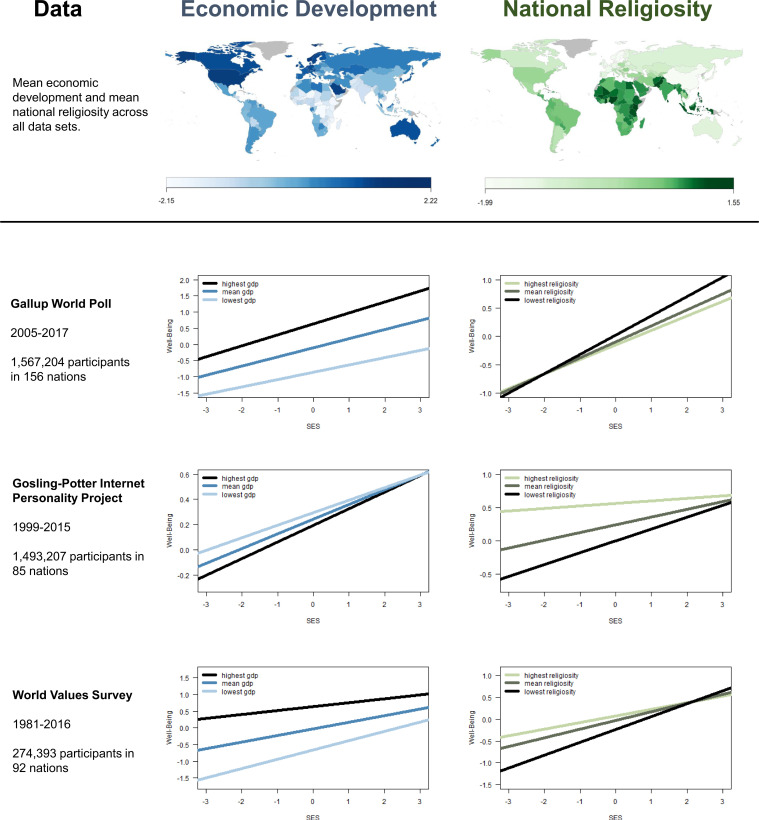
Distribution of national economic development, national religiosity, and estimated means of the cross-level interactions (model 3). The top row depicts mean national economic development and mean national religiosity worldwide (z-standardized and averaged across data sets). Lighter colors represent lower values, darker colors higher values. The three bottom rows depict estimated marginal means of the cross-level interactions when both national moderators were included in the model (i.e., model 3). Depicted are the moderating effects of national economic development and national religiosity on the association between SES and well-being in all three data sets.

Third, we tested the core hypothesis that national religiosity accounts for the attenuated effect of lower SES on well-being in developing nations. To this end, we combined models 1 and 2. Specifically, well-being was the criterion, and SES (level 1), national economic development (level 2), national religiosity (level 2), and the two cross-level interactions were the predictors (model 3). Fig. 2 displays the results of model 3. The top row depicts the distribution of national economic development and national religiosity across the world. Beneath those depictions, the right column shows that the psychological burden of lower SES was attenuated in more religious nations and that this finding emerged consistently in all three data sets: GWP: β = −0.02, 95% CI: −0.04 to −0.01; IPP: β = −0.03, 95% CI: −0.04 to −0.03; WVS: β = −0.03, 95% CI: −0.05 to −0.01. In sharp contrast, the left column shows that such consistency did not emerge for the cross-level interaction between SES and national economic development. In one data set, the psychological burden of lower SES was stronger in developed nations: GWP: β = 0.03, 95% CI: 0.01 to 0.04. In another data set, that psychological burden did not significantly vary as a function of national economic development: IPP: β = 0.01, 95% CI: −0.002 to 0.02. And in the final data set, the psychological burden of lower SES was weaker in developed nations: WVS: β = −0.03, 95% CI: −0.05 to −0.01.

The results of models 1 through 3 suggest that national religiosity can explain why the psychological burden of lower SES is amplified in developed nations (30). This is the case because the effect of national economic development on the psychological burden of lower SES (model 1) was reduced when we statistically accounted for national religiosity (model 3). A formal test of that reduction [model 4 (35); see *SI Appendix*, Fig. S1] revealed its significance: GWP: β = 0.02, 95% CI: 0.01 to 0.02; IPP: β = 0.01, 95% CI: 0.01 to 0.02; WVS: β = 0.01, 95% CI: 0.01 to 0.02. Indeed, national religiosity accounted for much of the apparent effect of national economic development on the psychological burden of lower SES: GWP: 42.4%; IPP: 56.0%; WVS: 56.8%.

Consistent with extant evidence, national economic development and national religiosity were strongly associated with five national covariates: collectivism, income inequality, pathogen prevalence, employment in agriculture, and percent of the population living in urban areas (see *Materials and Methods* and *SI Appendix*, Table S2). We tested whether our results persist when accounting for these national covariates. Specifically, we ran 15 variations of model 4 (5 covariates × 3 data sets). In each variation, we added another national covariate to the model (i.e., main effect and cross-level interaction with SES). In all 15 variations of model 4, national religiosity significantly accounted for the effect of national economic development on the psychological burden of lower SES (*SI Appendix*, Table S3). Thus, there is firm evidence that national religiosity is key to understanding why lower SES is less harmful to well-being in developing nations than it is in developed nations.

## Discussion

Lower SES is harmful to psychological well-being, an effect that has huge costs for the economy and society, and, above all, it constitutes a pressing humanitarian problem (4–7). Politicians, social scientists, and the general public have all long hoped that the harmful effect of lower SES would vanish with the rising economic development of nations (8, 9). Mounting evidence, however, quashed this hope; in fact, the economic development of nations even appeared to amplify the burden of SES, not reduce it. Here, we tested whether national religiosity can explain this counterintuitive discovery. National religiosity suggested itself as an explanation for two reasons. First, national economic development is a strong (inverse) associate of national religiosity (21, 22). Second, world religions uphold norms that, in part, function to ease the burden of lower SES (16, 29). In three large-scale, world-wide data sets, we received consistent evidence for this religiosity-based explanation.

We note three avenues for future research. First, the role of national religiosity in diminishing the psychological burden of lower SES was highly consistent across the three data sets. Yet, the role of national economic development was not. Specifically, even when national religiosity was not part of the model (model 1), national economic development did not qualify the psychological burden of lower SES in the WVS (but only in the WVS). Moreover, once we controlled for national religiosity in the WVS (model 3), the psychological burden of lower SES was more severe in developing nations. Future research may want to illuminate those inconsistencies between data sets, especially in light of our consistent findings regarding national religiosity.

Second, large-scale, cross-national surveys typically measure their constructs by means of self-report (i.e., use of subjective measures). In the present research, measurements of SES and psychological well-being were no exception. In all three data sets, participants categorized themselves into SES groups and rated their own well-being. Thus, replication attempts may want to circumvent self-report measures, using more objective measures instead.

Finally, the Western world has witnessed a marked decline in national religiosity over the last decades (36, 37). Viewed through the lens of the present research, this decline suggests that the harmful effects of lower SES on well-being should be more severe now than they were in the past. There is reason to believe that religious decline may even accelerate in the decades to come (38, 39). As a result, lower SES may well exert particularly harmful effects on well-being in the future. These hypotheses await empirical scrutiny. Nonetheless, the present results suggest that social scientists and policymakers should take note of the dwindling levels of national religiosity and the possibility that the harmful effects of lower SES will rise further as a result. The challenge will be to find alternatives to national religiosity to curb those harmful effects. Such alternatives will not be easily found because national religiosity exerts particularly powerful effects (40, 41). The anticipated difficulty of finding alternatives to national religiosity suggests that the search for such alternatives should start sooner rather than later and it should be a collective effort by social scientists and policymakers.

In all, we found evidence that national religiosity helps explain why the psychological burden of lower SES is attenuated in developing nations and amplified in developed ones. As such, our results demonstrate that the harmful effects of lower SES on well-being are not set in stone. In some of the most religious nations, we even found that those harmful effects were absent altogether.

## Materials and Methods

### Data Sets.

We used three data sets: the GWP [2005 through 2017 (32)], the IPP [1999 through 2015 (33)], and the WVS [1981 through 2016 (34)]. All data were collected following ethical clearance by either an organizational ethical review board (GWP and WVS) or the institutional review boards at the University of California and the University of Texas (IPP) and the informed consent of each participant. We used all available data from those data sets, but excluded participants who had missing values on any variable in our statistical models (listwise deletion). To assure a sufficiently precise estimation of associations within each nation, we followed the convention to exclude nations with less than 300 participants (42, 43). In effect, our main-text analyses rested on the following sample sizes. GWP: 1,567,204 participants in 156 nations; IPP: 1,493,207 participants in 85 nations; WVS: 274,393 participants in 92 nations.*SI Appendix, Table S1 summarizes the descriptive statistics for each nation in each data set (for more extensive descriptions of all three data sets, see refs. 21, 34, 43).

### Measures.

Typically, each data set included one measure per variable of interest for our investigation. In some instances, however, a data set included alternative measures. In those instances, we chose the single most suitable measure (our choices and their alternatives are explained in the following).

#### SES.

GWP: “Which one of these phrases comes closest to your own feelings about your household’s income these days: living comfortably on present income [1], getting by on present income [2], finding it difficult on present income [3], or finding it very difficult on present income [4]?” (reverse coded); IPP: “[…] where would you place yourself on the following spectrum for social class?” (working class [1], lower class [2], middle class [3], upper-middle class [4], upper class [5]); WVS: “People sometimes describe themselves as belonging to the working class, the middle class, or the upper or lower class. Would you describe yourself as belonging to the upper class [1], upper middle class [2], lower middle class [3], working class [4], lower class [5]” (reverse coded).^†^

#### Well-Being.

GWP: “Please imagine a ladder, with steps numbered from 0 at the bottom to 10 at the top. The top of the ladder represents the best possible life for you and the bottom of the ladder represents the worst possible life for you. On which step of the ladder would you say you personally feel you stand at this time?” (44); IPP: “I see myself as someone who has high self-esteem” (strongly disagree [1], strongly agree [5]; 45); WVS: “All things considered, how satisfied are you with your life as a whole these days? Using this card on which 1 means you are completely dissatisfied and 10 means you are completely satisfied where would you put your satisfaction with your life as a whole?” (46).^‡^

#### National Economic Development.

Following standard practice (47), we measured a nation’s economic development using that nation’s per capita gross domestic product [GDP in purchasing power parity (48)]. For each nation in each data set, we averaged the GDP across all years included in the data set, and we weighted yearly GDP according to the share of participants that were surveyed during this year. Following a standard economic method (15), we log-transformed (log10) the GDP data.

#### National Religiosity.

Following standard practice (42, 43), we averaged person-level religiosity within each nation for each data set. The religiosity items were as follows. GWP: “Is religion an important part of your daily life?” [yes [1], no [0] (21)]; IPP: “I see myself as someone who is very religious” [strongly disagree [1], strongly agree [5] (49)]; WVS (mean of the following four items [z-standardized]): “Independently of whether you go to church or not, would you say you are a religious person?” (religious [1], not religious [0], atheist [0]), “Do you believe in God?” (yes [1], no [0]), “Apart from weddings and funerals, about how often do you attend religious services these days?” (more than once a week [1] to never, practically never [8]; reverse coded), “Do you take some moments of prayer, meditation or contemplation or something like that?” (yes [1], no [0]). The three indices were near-perfectly interrelated (*SI Appendix*, Table S2).

#### Covariates.

We sought to assure that results are not spurious due to related but conceptually different national variables. Thus, as covariates, we assessed collectivism [meta-analytic update of Hofstede’s original index; reverse coded (50)], pathogen prevalence [nonzoonotic parasite prevalence (51)], income inequality [Gini coefficient (52)], employment in agriculture [percentage of total employment (52)], and the population living in urban areas [percentage of the total population (52)].

### Statistical Modeling.

We accounted for the nested data structure (persons nested in nations) by using linear mixed-effects models in R [mixed-effects model package *lme4* version 1.1-23, models 1 through 3 (53); mixed-effects path model package *lavaan* version 0.6-7, model 4 (54)]. We z-standardized all variables to obtain standardized coefficients (55), and group-mean centered all person-level predictors to unequivocally interpret our cross-level interactions (56). All our models included random intercepts (models 1 through 4) and, whenever possible, they also included random slopes for all person-level predictors [models 1 through 3 (57)]. All models included age and gender as control variables at the person-level, but the results without those controls were conceptually identical.

## Supplementary Material

Supplementary File

## Data Availability

In the current study, three large-scale data sets were used. For all data sets, all relevant national variables, including control variables, are reported in the *SI Appendix*. The GWP data that support the findings of this study are publicly available (https://www.gallup.com), but restrictions apply to the availability of these data, which were used under license for the current study. The data from the IPP that we analyzed in this study can be obtained from the first author upon request. The WVS data are publicly available at https://www.worldvaluessurvey.org. Analysis scripts and results can be retrieved from Open Science Framework, https://osf.io/ur6q2/?view_only=a7f2ff4a5e9a47f7b1f02eccbbc2a6ac.

## Footnotes

*Some of the robustness checks reported in the supplementary materials rested on somewhat smaller samples. This is the case because the robustness checks controlled for national covariates and some of those covariates had missing values. The supplementary materials report its results in tables, and those tables include the sample sizes on which the results rest.

^†^In addition to the chosen SES measures, all three data sets assessed highest education, and the GWP also assessed financial hardship and household income. We favor the chosen SES measures for several reasons: An independent validation study (*N* = 526 MTurk workers) revealed that the chosen measures correlated strongly with the McArthur Scale (58), the gold-standard measure of SES (GWP: *r* = 0.50, 95% CI: 0.43 to 0.56; IPP and WVS: *r* = 0.75, 95% CI: 0.71 to 0.79). Highest education, by contrast, was a comparatively weak correlate of the McArthur Scale; *r* = 0.34, 95% CI: 0.26 to 0.41. In line with this result, extant research on cross-national differences in the psychological burden of lower SES typically refrained from using highest education as an indicator of SES (1, 2, 8–10). In fact, there is no theoretical reason to expect that national religiosity can explain cross-national differences in the effects of little education on well-being (if there are any such cross-national differences). This is the case because religious norms do not lighten the stigma associated with little education or cast a bad light on much education. Financial hardship was also a comparatively weak correlate of the McArthur Scale (re food: *r* = 0.18, 95% CI: 0.10 to 0.26; re shelter: *r* = 0.11, 95% CI: 0.02 to 0.19) and financial hardship was available in only one of our three data sets. Finally, household income was a comparatively moderate correlate of the McArthur Scale (*r* = 0.51, 95% CI: 0.45 to 0.57), but it, too, was available in only one of our three data sets. Moreover, household income in the GWP is imprecise because it does not allow researchers to control appropriately for household size. Additionally, people often lack sufficiently accurate knowledge of their household income and are particularly hesitant to report their true household income (59). In line with those issues, we did not find that national religiosity moderated the effects of lower household income on well-being, even though extant research revealed that national religiosity moderated the effects of lower personal income on well-being (16).

^‡^In addition to those well-being indicators, the GWP assessed positive and negative emotions, and the IPP assessed anxious and depressed personality [as facets of neurotic personality (60)]. We did not include the results of those additional indicators in the main text because they assess transient states [positive and negative emotions (61)] or stable traits [anxious and depressed personality (60)]. For completeness reasons, *SI Appendix*, Tables S4 and S5 include the results involving those indicators, which were similar to the results reported in the main text.

## References

[1] R. Cummins, Personal income and subjective well-being: A review. J. Happiness Stud. 1, 133–158 (2000).

[2] B. Stevenson, J. Wolfers, Subjective well-being and income: Is there any evidence of satiation? Am. Econ. Rev. 103, 598–604 (2013).

[3] N. Goldman, D. A. Glei, M. Weinstein, Declining mental health among disadvantaged Americans. Proc. Natl. Acad. Sci. U.S.A. 115, 7290–7295 (2018). 29915079 10.1073/pnas.1722023115 PMC6048554

[4] A. V. Banerjee, A. F. Newman, Poverty, incentives, and development. Am. Econ. Rev. 84, 211–215 (1994).

[5] T. A. Kohler., Greater post-Neolithic wealth disparities in Eurasia than in North America and Mesoamerica. Nature 551, 619–622 (2017). 29143817 10.1038/nature24646 PMC5714260

[6] J. R. Platt, Extreme Poverty Is a Humanitarian Crisis—And an Environmental One (Revel, 2017).

[7] R. Narula, S. Saha, M. Koley, Warming will hit the poorest first. Nature 533, 440 (2016).

[8] R. A. Easterlin, Will raising the incomes of all increase the happiness of all? J. Econ. Behav. Organ. 27, 35–47 (1995).

[9] R. Veenhoven, Is happiness relative? Soc. Indic. Res. 24, 1–34 (1991).

[10] R. T. Howell, C. J. Howell, The relation of economic status to subjective well-being in developing countries: A meta-analysis. Psychol. Bull. 134, 536–560 (2008). 18605819 10.1037/0033-2909.134.4.536

[11] E. Diener, R. Biswas-Diener, Will money increase subjective well-being? Soc. Indic. Res. 57, 119–169 (2002).

[12] L. Tay, E. Diener, Needs and subjective well-being around the world. J. Pers. Soc. Psychol. 101, 354–365 (2011). 21688922 10.1037/a0023779

[13] M. Weber, The Protestant Ethic and the Spirit of Capitalism (1930) [trans.T. Parsons, Allen Unwin., London].

[14] E. Diener, M. Diener, C. Diener, Factors predicting the subjective well-being of nations. J. Pers. Soc. Psychol. 69, 851–864 (1995). 7473035 10.1037//0022-3514.69.5.851

[15] E. Diener, W. Ng, J. Harter, R. Arora, Wealth and happiness across the world: Material prosperity predicts life evaluation, whereas psychosocial prosperity predicts positive feeling. J. Pers. Soc. Psychol. 99, 52–61 (2010). 20565185 10.1037/a0018066

[16] J. E. Gebauer, A. D. Nehrlich, C. Sedikides, W. Neberich, The psychological benefits of income are contingent on individual-level and culture-level religiosity. Soc. Psychol. Personal. Sci. 4, 569–578 (2013).

[17] L. Tay, M. Morrison, E. Diener, Living among the affluent: Boon or bane? Psychol. Sci. 25, 1235–1241 (2014). 24756767 10.1177/0956797614525786

[18] C. A. Fulmer., On “feeling right” in cultural contexts: How person-culture match affects self-esteem and subjective well-being. Psychol. Sci. 21, 1563–1569 (2010). 20876880 10.1177/0956797610384742

[19] J. E. Gebauer, C. Sedikides, W. Neberich, Religiosity, social self-esteem, and psychological adjustment: On the cross-cultural specificity of the psychological benefits of religiosity. Psychol. Sci. 23, 158–160 (2012). 22222220 10.1177/0956797611427045

[20] M. Rosenberg, Society and the Adolescent Self-Image (Princeton University Press, 1965).

[21] M. Joshanloo, J. E. Gebauer, Religiosity’s nomological network and temporal change: Introducing an extensive country-level religiosity index based on Gallup World Poll data. Eur. Psychol. 25, 26–40 (2020).

[22] G. Saucier., Cross-cultural differences in a global “survey of world views.” J. Cross Cult. Psychol. 46, 53–70 (2015).

[23] E. Durkheim, The Elementary Forms of Religious Life (Free Press, 1965).

[24] J. Graham, J. Haidt, Beyond beliefs: Religions bind individuals into moral communities. Pers. Soc. Psychol. Rev. 14, 140–150 (2010). 20089848 10.1177/1088868309353415

[25] J. Henrich, The evolution of costly displays, cooperation and religion: Credibility enhancing displays and their implications for cultural evolution. Evol. Hum. Behav. 30, 244–260 (2009).

[26] M. Tomasello, The Cultural Origins of Human Cognition (Harvard University Press, 2009).

[27] S. Atran, J. Henrich, The evolution of religion: How cognitive by-products, adaptive learning heuristics, ritual displays, and group competition generate deep commitments to prosocial religions. Biol. Theory 5, 18–30 (2010).

[28] J. Marcus, K. V. Flannery, The coevolution of ritual and society: New 14C dates from ancient Mexico. Proc. Natl. Acad. Sci. U.S.A. 101, 18257–18261 (2004). 15601758 10.1073/pnas.0408551102 PMC539816

[29] J. E. Gebauer, C. Sedikides, Cultural religiosity: A neglected but powerful dimension of culture. Curr. Opin. Psychol. 40, 73–78 (2020). 33027746 10.1016/j.copsyc.2020.08.027

[30] V. Yzerbyt, D. Muller, C. Batailler, C. M. Judd, New recommendations for testing indirect effects in mediational models: The need to report and test component paths. J. Pers. Soc. Psychol. 115, 929–943 (2018). 30550319 10.1037/pspa0000132

[31] J. H. Jung, Country-level differences in the effects of financial hardship on life satisfaction: The role of religious context and age-contingent buffering. Soc. Ment. Health 8, 123–140 (2018).

[32] The Gallup Organization, Gallup World Poll (2020). www.gallup.com. Accessed 4 May 2018.

[33] S. D. Gosling, S. Vazire, S. Srivastava, O. P. John, Should we trust web-based studies? A comparative analysis of six preconceptions about internet questionnaires. Am. Psychol. 59, 93–104 (2004). 14992636 10.1037/0003-066X.59.2.93

[34] R. Inglehart., World values survey: All Rounds – Country-Pooled Datafile. Madrid, Spain & Vienna, Austria: JD Systems Institute & WVSA Secretariat. http://www.worldvaluessurvey.org/WVSDocumentationWVL.jsp. Accessed 8 January 2019.

[35] D. A. Kenny, D. Kashy, N. Bolder, “Data analysis in social psychology” in Handbook of Social Psychology, D. Gilbert, S. Fiske, G. Lindzey, Eds. (McGraw-Hill, ed. 4, 1998), pp. 233–265.

[36] D. J. Ruck, R. A. Bentley, D. J. Lawson, Religious change preceded economic change in the 20th century. Sci. Adv. 4, eaar8680 (2018). 30035222 10.1126/sciadv.aar8680 PMC6051740

[37] C. Murray, Coming Apart: The State of White America, 1960–2010 (Crown Forum, 2012).

[38] O. Ilany, Thank you god, we’ll take it from here: Why the world is becoming more secular. *Haaretz*, 26 September 2020.

[39] G. Bullard, The world’s newest major religion: No religion. *National Geographic News*, 22 April 2016.

[40] A. Norenzayan, Big Gods: How Religion Transformed Cooperation and Conflict (Princeton University Press, 2013).

[41] A. Norenzayan., The cultural evolution of prosocial religions. Behav. Brain Sci. 39, e1 (2016). 26785995 10.1017/S0140525X14001356

[42] J. E. Gebauer., The religiosity as social value hypothesis: A multi-method replication and extension across 65 countries and three levels of spatial aggregation. J. Pers. Soc. Psychol. 113, e18–e39 (2017). 27442765 10.1037/pspp0000104

[43] T. M. Entringer., Big Five facets and religiosity: Three large-scale, cross-cultural, theory-driven, and process-attentive tests. J. Pers. Soc. Psychol. 120, 1662–1695 (2021). 33119387 10.1037/pspp0000364

[44] H. Cantril, The Pattern of Human Concerns (Rutgers University Press, 1965).

[45] R. W. Robins, H. M. Hendin, K. H. Trzesniewski, Measuring global self-esteem: Construct validation of a single-item measure and the Rosenberg self-esteem scale. Pers. Soc. Psychol. Bull. 27, 151–161 (2001).

[46] F. Cheung, R. E. Lucas, Assessing the validity of single-item life satisfaction measures: Results from three large samples. Qual. Life Res. 23, 2809–2818 (2014). 24890827 10.1007/s11136-014-0726-4 PMC4221492

[47] J. M. Cypher, J. L. Dietz, The Process of Economic Development (Routledge, ed. 3, 2009).

[48] World Bank Group, GDP per capita, PPP (current international $). The World Bank (2019). https://data.worldbank.org/indicator/NY.GDP.PCAP.PP.CD. Accessed 3 May 2019.

[49] A. Norenzayan, I. G. Hansen, Belief in supernatural agents in the face of death. Pers. Soc. Psychol. Bull. 32, 174–187 (2006). 16382080 10.1177/0146167205280251

[50] V. Taras, P. Steel, B. L. Kirkman, Improving national cultural indices using a longitudinal meta-analysis of Hofstede’s dimensions. J. World Bus. 47, 329–341 (2012).

[51] C. L. Fincher, R. Thornhill, Parasite-stress promotes in-group assortative sociality: The cases of strong family ties and heightened religiosity. Behav. Brain Sci. 35, 61–79 (2012). 22289223 10.1017/S0140525X11000021

[52] United Nations Development Programme, Global Human Development Indicators, Human Develeopment Reports (2019). http://hdr.undp.org/en/countries. Accessed 14 June 2019.

[53] D. Bates, M. Mächler, B. Bolker, S. Walker, Fitting linear mixed-effects models using lme4. J. Stat. Softw. 67, 201–210 (2015).

[54] Y. Rosseel, Lavaan: An R package for structural equation modeling. J. Stat. Softw. 48 (2012).

[55] T. A. B. Snijders, R. J. Bosker, Multilevel Analysis: An Introduction to Basic and Advances Multilevel Modeling (Sage Publishers, ed. 2, 2012).

[56] C. K. Enders, D. Tofighi, Centering predictor variables in cross-sectional multilevel models: A new look at an old issue. Psychol. Methods 12, 121–138 (2007). 17563168 10.1037/1082-989X.12.2.121

[57] D. J. Barr, R. Levy, C. Scheepers, H. J. Tily, Random effects structure for confirmatory hypothesis testing: Keep it maximal. J. Mem. Lang. 68, 255–278 (2013). 10.1016/j.jml.2012.11.001 PMC3881361 24403724

[58] N. E. Adler, J. Stewart, The MacArthur Scale of Subjective Social Status (Mac Arthur Foundation & University of California, San Francisco, 2007).

[59] M. A. Diemer, R. S. Mistry, M. E. Wadsworth, I. López, F. Reimers, Best practices in conceptualizing and measuring social class in psychological research. Anal. Soc. Issues Public Policy 13, 77–113 (2013).

[60] C. J. Soto, O. P. John, Ten facet scales for the Big Five Inventory: Convergence with NEO PI-R facets, self-peer agreement, and discriminant validity. J. Res. Pers. 43, 84–90 (2009).

[61] E. Diener, S. Oishi, L. Tay, Advances in subjective well-being research. Nat. Hum. Behav. 2, 253–260 (2018). 30936533 10.1038/s41562-018-0307-6

