# Supplementary material for: National religiosity eases the psychological burden of poverty
Source: Proc Natl Acad Sci U S A. 2021 Sep 20;118(39):e2103913118. doi: 10.1073/pnas.2103913118 (PMC8488579; doi:10.1073/pnas.2103913118)
Supplement: Supplementary File [file pnas.2103913118.sapp.pdf]

PNAS
www.pnas.org

Supplementary Information for

National religiosity eases the psychological burden of poverty

Authors: Jana B. Berkessel[1*†], Jochen E. Gebauer[1,2], Mohsen Joshanloo[3], Wiebke Bleidorn[4], Peter J. Rentfrow[5], Jeff Potter[6], Samuel D. Gosling[7,8]

Affiliations:

[1]University of Mannheim.

[2]University of Copenhagen.

[3]Keimyung University.

[4]University of Zurich.

[5]University of Cambridge.

[6]Atof Inc.

[7]University of Texas at Austin.

[8]University of Melbourne.

This PDF file includes:

Tables S1 to S5

Figure S1

SI References

Country	GWP							IPP							WVS						
	N	Age		%	Nat.	GDP	Corr.	*N*	Age		%	Nat.	GDP	Corr.	*N*	Age		%	Nat.	GDP	Corr.
		M	*SD*	Women	Rel.		SES-WB		*M*	*SD*	Women	Rel.		SES-WB		*M*	*SD*	Women	Rel.		SES-WB
Afghanistan	11,413	33.02	13.06	49.35	0.97	3.24	0.30														
Albania	9,590	42.88	17.56	55.46	0.52	4.02	0.47	303	27.85	10.43	64.03	2.64	3.98	0.07	1,937	40.80	15.09	50.85	-0.07	3.58	0.42
Algeria	7,856	36.48	14.89	49.83	0.93	4.14	0.43								2,290	36.53	14.32	48.91	0.15	4.06	0.27
Angola	3,328	32.75	13.77	47.48	0.87	3.83	0.36														
Argentina	10,722	45.55	18.66	60.48	0.63	4.28	0.32	36,184	28.94	9.60	66.69	2.38	4.24	0.13	5,101	42.35	17.18	52.54	-0.01	4.08	0.04
Armenia	10,615	45.25	18.55	60.46	0.72	3.89	0.33	324	29.90	12.55	65.12	2.42	3.82	0.01	3,026	41.06	17.34	57.57	-0.00	3.57	0.31
Aruba								357	33.77	11.93	66.11	2.58	4.56	0.17							
Australia	10,948	53.14	18.04	56.81	0.32	4.64	0.40	31,881	30.10	11.45	60.83	2.11	4.60	0.17	4,673	48.21	17.59	53.69	-0.42	4.49	0.17
Austria	10,670	49.17	16.53	55.91	0.46	4.67	0.37	13,114	31.85	11.52	58.74	2.08	4.63	0.19							
Azerbaijan	10,367	39.13	15.21	51.63	0.42	4.20	0.40								2,939	37.87	14.21	50.80	-0.05	3.85	0.27
Bahamas								308	29.01	12.13	70.45	3.08	4.47	-0.02							
Bahrain	12,832	35.75	12.69	41.99	0.95	4.63	0.38														
Bangladesh	13,923	36.81	15.04	51.36	0.98	3.44	0.37	364	28.30	12.12	36.54	2.89	3.42	0.11	2,914	34.63	11.65	44.06	0.51	3.10	0.27
Belarus	10,612	44.89	17.72	59.46	0.35	4.23	0.29								3,536	44.21	16.82	56.53	-0.42	3.94	0.29
Belgium	10,642	49.14	16.70	55.00	0.33	4.63	0.39	18,140	32.22	11.36	61.54	1.73	4.60	0.19							
Belize	859	35.25	14.25	52.97	0.65	3.90	0.16														
Benin	7,392	34.36	14.74	46.69	0.87	3.31	0.23														
Bhutan	2,907	37.65	15.32	46.16	0.97	3.89	0.20														
Bolivia	10,351	39.02	17.22	57.34	0.88	3.78	0.25	2,360	27.83	8.06	56.48	2.86	3.71	0.15							
Bosnia															787	41.21	14.34	41.80	0.02	3.58	0.24
Bosnia Herzegovina	10,538	45.22	17.91	55.24	0.71	4.01	0.43								1,169	40.68	15.24	51.75	0.07	3.67	0.28
Botswana	8,637	36.14	16.11	59.21	0.88	4.18	0.33														
Brazil	11,824	43.30	17.57	60.43	0.89	4.17	0.26	3,915	31.79	10.46	50.70	2.56	4.18	0.14	4,075	39.88	15.61	57.42	0.31	4.03	0.09
Bulgaria	9,732	52.75	18.05	59.86	0.39	4.22	0.51	518	27.38	8.52	60.04	2.24	4.17	0.15	1,879	46.60	17.00	53.01	-0.56	3.92	0.35
Burkina Faso	9,358	34.00	14.25	42.21	0.91	3.19	0.22								1,267	34.33	13.80	46.65	0.52	3.10	0.24
Burundi	3,787	34.94	14.56	49.91	0.94	2.86	0.26														

Country	GWP							IPP							WVS						
	N	Age		%	Nat.	GDP	Corr.	*N*	Age		%	Nat.	GDP	Corr.	*N*	Age		%	Nat.	GDP	Corr.
		M	*SD*	Women	Rel.		SES-WB		*M*	*SD*	Women	Rel.		SES-WB		*M*	*SD*	Women	Rel.		SES-WB
Cambodia	12,147	39.10	15.27	63.43	0.87	3.47	0.33														
Cameroon	10,638	32.86	13.96	49.04	0.94	3.50	0.28														
Canada	12,564	50.19	17.25	52.97	0.41	4.63	0.42	64,094	29.66	11.56	61.15	2.18	4.61	0.14	3,912	47.20	17.61	59.07	-0.03	4.53	0.12
Central African Republic	4,708	33.04	13.18	49.17	0.97	2.90	0.26														
Chad	10,337	32.35	13.07	41.48	0.93	3.29	0.19														
Chile	10,965	46.36	18.05	58.71	0.68	4.31	0.35	15,593	30.91	10.75	62.62	2.61	4.24	0.10	4,126	41.96	16.14	52.81	-0.00	4.05	0.18
China	48,841	44.83	16.23	53.78	0.16	4.07	0.42	4,414	29.50	7.71	58.20	2.37	3.96	0.10	6,259	42.39	13.95	50.06	-1.31	3.80	0.27
Colombia	10,567	42.84	17.60	63.89	0.87	4.08	0.35	12,250	28.17	8.70	64.02	2.89	4.02	0.09	7,394	36.93	13.31	49.22	0.31	3.89	0.05
Comoros	6,756	37.06	16.94	57.12	0.98	3.38	0.32														
Congo Brazzaville	7,020	36.07	14.36	46.34	0.90	3.74	0.26														
Congo Kinshasa	7,504	32.46	13.07	43.24	0.94	2.89	0.29														
Costa Rica	10,531	41.22	17.81	54.21	0.82	4.15	0.29	2,509	28.70	8.84	62.85	2.79	4.10	0.11							
Croatia	9,338	44.02	17.42	56.55	0.64	4.34	0.42	805	28.22	8.09	59.63	2.36	4.30	0.15	1,138	43.09	16.39	52.28	-0.10	3.94	0.24
Cyprus	8,229	46.52	17.86	54.55	0.73	4.51	0.48	451	31.56	10.13	61.86	2.56	4.50	0.15	2,022	41.81	16.74	52.47	-0.06	4.50	0.11
Czech Republic	9,759	46.78	16.96	57.70	0.25	4.49	0.39	546	29.02	9.65	45.42	2.09	4.45	0.05	1,110	47.75	16.64	56.58	-0.87	4.13	0.29
Denmark	11,559	50.41	17.36	57.30	0.19	4.66	0.30	3,794	29.88	10.53	50.40	1.75	4.63	0.17							
Djibouti	4,796	32.69	12.82	46.91	0.96	3.43	0.30														
Dominican Republic	10,522	41.70	17.84	58.34	0.88	4.09	0.30	2,379	27.76	8.35	69.99	3.16	4.02	0.12	401	28.66	8.88	59.35	0.15	3.70	0.18
Ecuador	10,725	41.41	17.70	61.25	0.86	4.01	0.32	3,993	30.35	9.74	60.43	2.94	3.99	0.03	1,200	39.81	16.14	51.58	0.22	4.04	0.15
Egypt	19,422	38.88	15.20	47.88	0.98	4.00	0.33	1,160	27.06	8.73	57.84	3.24	3.97	0.08	7,463	39.71	14.91	57.91	0.27	3.90	0.11
El Salvador	10,326	38.00	16.39	53.51	0.87	3.83	0.30	1,434	29.51	8.38	59.14	3.08	3.79	0.09	1,141	37.52	15.54	51.80	0.25	3.64	0.15
Estonia	8,915	50.93	18.47	61.39	0.20	4.43	0.44	375	27.51	9.30	57.07	1.82	4.36	0.18	2,444	46.62	17.34	55.61	-0.89	4.24	0.35
Ethiopia	6,269	32.80	13.70	57.87	0.98	3.18	0.37								1,422	29.96	10.11	48.95	0.47	2.91	0.34
Finland	9,657	54.98	17.55	55.04	0.31	4.62	0.38	4,516	28.28	9.38	59.94	1.94	4.58	0.18	1,836	45.05	17.25	51.74	-0.34	4.37	0.20
France	12,548	48.62	17.05	56.85	0.29	4.58	0.36	3,459	32.58	10.73	55.39	1.94	4.56	0.14							
Gabon	6,735	35.73	14.30	46.33	0.89	4.24	0.32														
Gambia	881	36.22	16.40	60.39	0.96	3.23	0.06														

Country	GWP							IPP							WVS						
	N	Age		%	Nat.	GDP	Corr.	*N*	Age		%	Nat.	GDP	Corr.	*N*	Age		%	Nat.	GDP	Corr.
		M	*SD*	Women	Rel.		SES-WB		*M*	*SD*	Women	Rel.		SES-WB		*M*	*SD*	Women	Rel.		SES-WB
Georgia	10,664	47.51	18.26	56.30	0.81	3.91	0.46								4,612	43.24	17.14	54.14	0.18	3.72	0.27
Germany	26,515	51.57	18.16	53.72	0.39	4.63	0.44	93,222	33.67	11.68	56.87	2.01	4.60	0.18	5,722	48.13	17.33	53.36	-0.75	4.54	0.27
Ghana	10,175	34.62	14.68	49.02	0.94	3.55	0.27								3,074	32.39	13.47	49.64	0.62	3.49	0.20
Greece	9,850	49.60	18.60	55.44	0.71	4.44	0.42	1,703	29.95	9.21	63.53	2.23	4.43	0.16							
Guatemala	9,944	36.40	16.73	54.28	0.88	3.85	0.26	2,588	28.19	8.42	61.24	2.95	3.82	0.13							
Guinea	6,495	37.05	16.73	46.57	0.96	3.27	0.27														
Guyana	471	36.49	15.27	50.53	0.87	3.70	0.18														
Haiti	3,974	36.87	15.75	50.96	0.81	3.22	0.13								1,612	33.57	14.74	51.18	0.21	3.25	-0.03
Honduras	10,220	36.57	16.19	53.79	0.85	3.63	0.28	1,345	28.94	7.94	64.76	3.05	3.60	0.07							
Hong Kong	7,550	42.50	18.54	53.05	0.25	4.72	0.43	2,758	29.76	9.69	64.29	2.36	4.67	0.09	2,192	44.36	16.05	53.15	-0.92	4.65	0.22
Hungary	9,770	52.54	18.05	59.45	0.40	4.38	0.43	815	30.44	9.95	59.14	2.12	4.33	0.14	1,615	45.35	17.25	53.56	-0.59	4.22	0.32
Iceland	3,516	47.51	16.88	55.97	0.35	4.68	0.44	535	29.02	9.87	58.88	2.08	4.61	0.21							
India	45,476	36.42	14.65	45.01	0.83	3.70	0.35	13,181	27.49	7.59	41.76	3.14	3.66	0.07	9,535	39.89	14.18	43.11	0.30	3.47	0.25
Indonesia	13,800	38.66	14.26	56.23	0.98	3.96	0.32	1,946	27.15	7.97	55.65	3.17	3.95	0.08	2,717	39.09	14.30	48.07	0.46	3.77	0.16
Iran	11,187	36.48	13.58	49.36	0.82	4.25	0.38	562	28.19	8.52	53.56	2.45	4.23	0.13	4,965	33.59	14.17	47.79	0.34	4.13	0.25
Iraq	14,346	35.16	12.35	44.91	0.85	4.15	0.25								6,058	36.96	13.65	50.50	-0.05	4.05	0.27
Ireland	10,103	47.08	15.98	53.96	0.55	4.73	0.36	8,632	29.48	10.18	61.98	2.21	4.66	0.16							
Israel	10,616	41.62	16.37	51.99	0.45	4.51	0.29	1,583	31.51	10.84	55.40	2.00	4.47	0.17							
Italy	12,359	49.05	16.17	57.25	0.65	4.56	0.37	3,174	33.28	10.50	52.80	2.26	4.54	0.12	924	45.67	15.55	49.24	0.20	4.48	0.23
Ivory Coast	5,769	34.20	13.38	39.28	0.90	3.52	0.26														
Jamaica	1,810	40.38	17.11	53.31	0.83	3.93	0.28	595	29.99	9.56	76.81	3.44	3.91	0.02							
Japan	14,838	53.65	16.77	53.38	0.25	4.57	0.42	2,775	29.13	9.20	51.93	2.24	4.55	0.11	5,424	47.94	15.98	52.08	-0.74	4.44	0.40
Jordan	14,616	35.27	14.75	51.18	0.94	3.96	0.36								3,579	37.78	14.89	50.63	0.33	3.90	0.23
Kazakhstan	9,944	41.81	16.48	58.54	0.45	4.35	0.28								1,500	40.02	15.35	60.40	-0.19	4.33	0.23
Kenya	11,875	32.41	12.51	51.15	0.94	3.42	0.24	598	30.68	8.09	62.21	3.50	3.42	0.06							
Kosovo	9,655	38.81	16.23	49.30	0.81	3.94	0.32														
Kuwait	12,594	35.69	11.95	37.91	0.91	4.88	0.23	388	28.91	9.53	58.51	3.08	4.90	0.02	1,089	36.59	11.77	34.89	0.07	4.87	0.19
Kyrgyzstan	10,458	41.43	17.12	61.91	0.70	3.49	0.29								2,513	38.06	14.78	52.85	0.09	3.40	0.11

Country	GWP							IPP							WVS						
	N	Age		%	Nat.	GDP	Corr.	*N*	Age		%	Nat.	GDP	Corr.	*N*	Age		%	Nat.	GDP	Corr.
		M	*SD*	Women	Rel.		SES-WB		*M*	*SD*	Women	Rel.		SES-WB		*M*	*SD*	Women	Rel.		SES-WB
Laos	5,599	37.55	14.50	56.89	0.96	3.64	0.28														
Latvia	8,596	47.37	18.37	61.37	0.36	4.35	0.39	312	26.68	6.74	58.97	2.01	4.28	0.19	1,159	42.30	15.89	55.05	-0.38	3.76	0.32
Lebanon	13,789	38.59	15.44	52.25	0.85	4.18	0.33	679	26.89	7.90	55.38	2.93	4.17	0.08	1,184	38.39	14.87	51.01	0.19	4.17	0.19
Lesotho	2,714	41.60	19.85	59.58	0.95	3.45	0.24														
Liberia	6,143	33.70	13.53	51.39	0.94	3.07	0.26														
Libya	3,893	34.03	11.11	37.19	0.97	4.28	0.25								2,070	38.34	13.42	48.74	0.25	4.24	0.16
Lithuania	8,415	47.06	18.02	55.66	0.41	4.39	0.46								971	43.67	16.82	49.85	0.02	3.85	0.37
Luxembourg	8,813	46.87	16.26	53.01	0.35	4.99	0.29	402	33.18	12.33	57.46	1.87	4.94	0.16							
Macedonia															1,974	41.35	15.07	47.87	-0.06	3.76	0.27
Madagascar	7,752	37.23	15.32	54.44	0.93	3.16	0.23														
Malawi	8,604	33.64	14.84	59.76	0.98	3.03	0.22														
Malaysia	10,533	36.87	14.32	51.29	0.90	4.34	0.27	5,220	26.52	8.17	63.49	3.02	4.34	0.08	2,458	36.43	13.49	49.23	0.30	4.31	0.05
Mali	9,440	35.89	15.09	45.40	0.95	3.29	0.18								1,115	36.86	14.61	48.52	0.58	3.23	0.29
Malta	8,856	48.54	17.63	57.15	0.84	4.52	0.41														
Mauritania	13,079	34.63	14.09	44.35	0.97	3.54	0.28														
Mauritius	3,876	45.60	16.93	55.13	0.94	4.30	0.31														
Mexico	10,577	40.51	16.04	51.96	0.63	4.21	0.28	52,970	27.96	8.60	58.04	2.53	4.17	0.13	4,757	36.63	14.77	49.88	0.21	4.02	0.09
Moldova	10,398	42.65	16.74	56.75	0.73	3.65	0.35								2,897	42.81	16.37	53.30	0.12	3.38	0.32
Mongolia	9,601	39.50	16.11	56.99	0.45	4.00	0.34														
Montenegro	9,454	42.39	16.58	52.85	0.58	4.18	0.47								1,136	43.45	15.30	49.03	-0.33	3.83	0.39
Morocco	9,535	38.61	15.71	54.31	0.95	3.86	0.43								3,440	35.74	12.84	50.12	0.34	3.73	0.31
Mozambique	4,574	32.44	14.09	50.33	0.90	3.00	0.23														
Myanmar	6,529	43.64	15.86	61.60	0.95	3.72	0.28														
Namibia	2,749	34.87	15.59	61.00	0.91	3.98	0.24														
Nepal	11,451	37.19	15.59	57.52	0.95	3.33	0.28														
Netherlands	10,519	50.79	16.47	52.73	0.31	4.68	0.38	77,066	32.99	11.92	63.45	1.93	4.65	0.17	1,865	53.61	16.29	53.40	-0.74	4.65	0.19
New Zealand	9,480	51.34	18.15	58.11	0.32	4.55	0.41	8,754	31.53	12.27	62.85	2.12	4.49	0.14	2,525	48.72	16.60	54.89	-0.55	4.40	0.20
Nicaragua	10,250	35.88	15.46	53.17	0.85	3.66	0.29	1,063	29.60	8.63	67.17	3.04	3.61	0.06							

Country	GWP							IPP							WVS						
	N	Age		%	Nat.	GDP	Corr.	*N*	Age		%	Nat.	GDP	Corr.	*N*	Age		%	Nat.	GDP	Corr.
		M	*SD*	Women	Rel.		SES-WB		*M*	*SD*	Women	Rel.		SES-WB		*M*	*SD*	Women	Rel.		SES-WB
Niger	10,101	33.88	14.68	44.41	0.97	2.94	0.24														
Nigeria	9,363	33.05	13.41	43.39	0.95	3.73	0.23	683	30.82	7.32	50.81	3.71	3.73	0.03	5,524	31.63	11.25	48.91	0.63	3.49	0.24
Norway	7,855	52.01	18.01	51.22	0.22	4.79	0.31	10,832	32.71	11.74	58.87	1.69	4.77	0.23	1,965	44.49	16.16	49.67	-0.70	4.61	0.14
Pakistan	17,084	34.52	14.00	50.04	0.94	3.65	0.34	2,341	26.26	7.98	40.15	3.33	3.64	-0.00							
Panama	10,445	41.00	17.26	53.84	0.83	4.27	0.24	1,045	30.69	9.56	65.36	3.13	4.18	0.05							
Paraguay	9,592	42.58	17.72	57.79	0.90	4.01	0.32	1,507	28.12	9.17	66.22	2.97	3.96	0.09							
Peru	10,304	40.20	17.28	57.20	0.83	4.04	0.29	9,844	29.22	9.06	56.32	2.86	3.98	0.12	5,230	36.80	14.42	50.21	0.26	3.85	0.14
Philippines	11,622	40.72	16.87	57.07	0.95	3.80	0.24	10,203	25.03	8.35	73.12	3.28	3.76	0.05	3,583	39.24	15.15	49.90	0.43	3.62	0.15
Poland	10,383	47.88	17.95	59.28	0.67	4.37	0.42	1,347	28.47	8.58	51.74	2.18	4.32	0.16	2,758	46.86	17.25	51.31	0.40	4.12	0.24
Portugal	10,659	48.48	17.13	56.53	0.62	4.45	0.42	1,146	31.60	10.01	53.14	1.98	4.42	0.20							
Puerto Rico	489	54.09	18.92	57.67	0.83	4.57	0.24	2,225	31.73	10.92	65.93	3.07	4.53	0.07	1,780	44.00	16.39	64.78	0.35	4.34	0.10
Qatar	6,544	33.04	10.92	39.61	0.95	5.09	0.30	300	31.49	9.50	55.33	3.10	5.09	0.18	1,045	37.72	12.80	54.16	0.51	5.09	0.13
Romania	9,667	52.53	18.58	57.53	0.82	4.29	0.47	1,704	28.15	7.91	63.50	2.50	4.23	0.13	4,235	46.69	17.13	54.26	0.26	4.07	0.31
Russia	23,760	44.75	17.86	64.31	0.33	4.36	0.34	827	27.99	8.64	59.01	2.28	4.32	0.17	4,166	46.37	16.87	56.82	-0.52	4.14	0.22
Rwanda	8,681	35.27	14.66	51.51	0.94	3.21	0.31								2,910	34.09	12.49	50.07	0.61	3.12	0.29
Saudi Arabia	15,523	32.64	11.02	45.78	0.96	4.69	0.26	761	30.78	9.23	40.47	3.17	4.68	0.13	1,428	32.44	10.48	49.09	0.13	4.55	0.20
Senegal	10,368	34.68	14.78	47.64	0.97	3.46	0.31														
Serbia	9,196	47.30	17.33	54.38	0.55	4.12	0.44	720	28.16	8.04	57.08	2.30	4.09	0.18	3,461	44.60	15.77	50.30	-0.21	3.84	0.31
Sierra Leone	8,095	35.34	15.63	49.97	0.97	3.14	0.18														
Singapore	11,204	43.45	16.08	52.10	0.62	4.88	0.27	6,786	25.97	8.20	62.16	2.62	4.86	0.13	3,324	38.81	16.12	53.82	-0.01	4.80	0.12
Slovakia	7,801	48.53	16.96	57.86	0.48	4.45	0.46								1,082	44.45	16.04	49.63	-0.05	4.03	0.27
Slovenia	9,287	51.16	17.35	57.94	0.37	4.49	0.45	386	29.57	9.23	53.11	1.84	4.45	0.21	2,910	46.40	17.17	54.40	-0.35	4.35	0.25
South Africa	11,552	36.05	14.95	53.64	0.85	4.09	0.29	4,923	31.85	10.31	64.31	2.97	4.07	0.12	11,850	37.99	14.95	49.86	0.30	3.94	0.32
South Korea	12,445	47.03	18.66	50.08	0.45	4.51	0.39	1,883	29.16	7.93	40.57	2.42	4.48	0.17	2,375	42.23	14.50	50.36	-0.40	4.25	0.27
South Sudan	3,357	33.05	14.17	51.03	0.92	3.28	0.15														
Spain	12,716	45.20	15.94	56.09	0.39	4.53	0.39	53,037	30.28	9.33	64.65	1.90	4.50	0.15	4,672	45.98	18.28	50.98	-0.37	4.36	0.17
Sri Lanka	9,934	43.11	16.34	60.17	0.99	3.99	0.19	340	29.28	9.64	51.76	3.34	3.97	0.07							

	GWP							IPP							WVS						
Country	*N*	Age		%	Nat.	GDP	Corr.	*N*	Age		%	Nat.	GDP	Corr.	*N*	Age		%	Nat.	GDP	Corr.
		M	*SD*	Women	Rel.		SES-WB		*M*	*SD*	Women	Rel.		SES-WB		*M*	*SD*	Women	Rel.		SES-WB
Sudan	7,378	35.55	13.91	46.04	0.91	3.56	0.23														
Suriname	434	39.23	14.65	53.69	0.79	4.19	0.31														
Sweden	11,464	50.91	17.97	53.97	0.19	4.65	0.32	7,993	30.22	11.05	57.85	1.67	4.63	0.19	2,873	46.63	17.71	50.02	-0.90	4.50	0.16
Switzerland	6,327	52.26	17.76	54.88	0.41	4.78	0.36	17,235	33.93	11.95	58.04	2.05	4.73	0.17	2,317	48.96	17.00	52.14	-0.18	4.55	0.19
Tajikistan	10,545	37.23	16.23	59.73	0.78	3.38	0.26														
Tanzania	10,356	34.65	14.03	50.36	0.96	3.37	0.24								1,073	37.67	13.52	44.08	0.56	3.11	0.13
Thailand	13,126	43.53	15.36	64.98	0.96	4.15	0.29	1,595	27.53	10.02	58.93	2.74	4.14	0.09	2,673	45.31	14.36	49.49	-0.36	4.13	0.07
Togo	5,650	34.04	14.16	45.01	0.86	3.16	0.27														
Trinidad and Tobago	1,893	42.29	17.31	52.46	0.85	4.50	0.26	632	30.78	9.96	72.31	3.08	4.49	0.10	1,964	44.09	17.59	54.84	0.31	4.46	0.14
Tunisia	11,940	38.16	16.02	49.53	0.92	4.03	0.46								1,197	38.72	16.16	47.45	-0.03	4.04	0.17
Turkey	12,526	37.39	14.50	49.45	0.80	4.33	0.34	992	29.37	8.70	57.86	2.39	4.27	0.08	7,974	36.94	13.88	49.84	0.12	4.06	0.21
Turkmenistan	7,579	36.81	15.32	50.30	0.73	4.15	0.09														
Uganda	10,346	32.43	13.31	48.74	0.95	3.21	0.22								847	30.35	9.26	51.59	0.51	2.94	0.12
Ukraine	10,542	46.08	17.66	62.98	0.43	3.91	0.37								4,997	45.53	16.96	60.84	-0.23	3.74	0.34
United Arab Emirates	16,071	34.51	11.05	44.84	0.94	4.81	0.31	2,571	30.28	9.45	53.95	2.97	4.81	0.16							
United Kingdom	22,175	50.61	16.67	53.45	0.30	4.58	0.39	80,779	29.42	10.79	54.54	1.90	4.56	0.14	1,081	46.07	18.78	53.75	-0.55	4.44	0.15
United States	12,803	52.25	18.35	50.78	0.65	4.72	0.44	745,859	30.07	11.45	62.95	2.70	4.70	0.13	7,168	45.34	17.34	52.06	0.15	4.56	0.20
Uruguay	10,640	49.15	19.55	61.02	0.43	4.27	0.37	2,534	31.41	10.63	68.47	2.19	4.19	0.16	2,904	45.92	18.14	55.89	-0.49	4.12	0.11
Uzbekistan	9,428	39.47	16.54	62.75	0.58	3.71	0.26								1,430	39.30	14.84	61.05	-0.22	3.65	0.13
Venezuela	9,373	42.79	17.30	60.55	0.78	4.24	0.36	7,302	29.12	9.66	66.90	2.93	4.21	0.10	2,355	36.48	14.42	49.60	0.18	4.05	0.15
Vietnam	11,266	42.34	15.62	54.64	0.34	3.70	0.33	469	28.13	8.80	59.91	2.48	3.67	0.06	2,445	41.45	15.76	49.37	-0.96	3.46	0.11
Yemen	12,539	34.28	13.95	49.41	0.97	3.58	0.45								975	35.58	13.17	50.36	0.26	3.60	0.27
Zambia	9,232	32.24	13.13	52.03	0.95	3.54	0.26								1,214	29.57	11.30	47.86	0.40	3.41	0.20
Zimbabwe	10,573	34.90	15.28	54.59	0.89	3.30	0.38								2,465	34.25	13.99	52.25	0.50	3.36	0.30

Table S1. National descriptive statistics of all datasets. Following previous research with the same datasets (1, 2) we included all regions that met our statistical requirements, even if they were not technically a nation. [a]In line with the documentation of the World Values Survey Bosnia and Bosnia-Herzegovina were treated as two entities, because the data from Bosnia were collected before Bosnia-Herzegovina was founded.

Variable	*M*	*SD*	1	2	3	4	5	6	7	8	9	10
1. National Religiosity - GWP	72.04	24.92										
2. National Religiosity - IPP	2.61	0.44	.75**<br>[.67, .81]									
3. National Religiosity - WVS	-0.05	0.44	.90**<br>[.85, .93]	.77**<br>[.68, .84]								
4. Log 10 GDP per capita - GWP	4.01	0.52	-.61**<br>[-.70, -.50]	-.46**<br>[-.57, -.33]	-.51**<br>[-.64, -.35]							
5. Log 10 GDP per capita - IPP	4.29	0.36	-.47**<br>[-.62, -.29]	-.57**<br>[-.70, -.41]	-.30*<br>[-.50, -.07]	1.00**<br>[1.00, 1.00]						
6. Log 10 GDP per capita - WVS	3.96	0.45	-.40**<br>[-.56, -.23]	-.47**<br>[-.61, -.30]	-.42**<br>[-.57, -.24]	.96**<br>[.93, .97]	.97**<br>[.95, .98]					
7. Individualism	-0.58	0.56	-.78**<br>[-.83, -.71]	-.62**<br>[-.71, -.51]	-.62**<br>[-.73, -.48]	.66**<br>[.56, .74]	.56**<br>[.38, .69]	.49**<br>[.32, .63]				
8. Income Inequality	37.64	7.99	.50**<br>[.37, .62]	.41**<br>[.26, .53]	.35**<br>[.16, .52]	-.39**<br>[-.52, -.24]	-.48**<br>[-.63, -.29]	-.19<br>[-.38, .02]	-.50**<br>[-.61, -.36]			
9. Pathogen Prevalence	0.05	1.92	.60**<br>[.49, .69]	.52**<br>[.40, .62]	.48**<br>[.32, .61]	-.68**<br>[-.75, -.58]	-.64**<br>[-.75, -.50]	-.49**<br>[-.62, -.32]	-.63**<br>[-.71, -.54]	.54**<br>[.42, .64]		
10. Employment in Agriculture	27.12	24.99	.57**<br>[.45, .66]	.36**<br>[.22, .49]	.52**<br>[.37, .65]	-.85**<br>[-.89, -.80]	-.75**<br>[-.83, -.64]	-.77**<br>[-.84, -.67]	-.58**<br>[-.67, -.47]	.31**<br>[.17, .45]	.61**<br>[.51, .69]	
11. Urban Population	59.07	22.57	-.50**<br>[-.61, -.38]	-.41**<br>[-.53, -.28]	-.42**<br>[-.56, -.25]	.79**<br>[.72, .84]	.67**<br>[.54, .77]	.78**<br>[.69, .85]	.44**<br>[.30, .55]	-.18*<br>[-.32, -.02]	-.35**<br>[-.47, -.23]	-.69**<br>[-.76, -.61]

Table S2. Means, standard deviations, and correlations with confidence intervals between national religiosity, national economic development, and the national covariates

Control variable included in model	GWP					IPP					WVS				
	beta	95% CI	% mediated	*N*	Nations	*beta*	95% CI	% mediated	*N*	Nations	*beta*	95% CI	% mediated	*N*	Nations
Individualism	.022	[.017, .027]	45.9%	1,374,821	136	.014	[.009, .020]	44.1%	1,441,228	76	.012	[.007, .018]	-33.2%	246,777	82
Income Inequality	.012	[.009, .014]	23.4%	1,415,345	136	.020	[.015, .024]	65.0%	1,473,708	73	.017	[.011, .023]	-91.8%	254,889	81
Pathogen Prevalence	.015	[.012, .019]	44.6%	1,554,203	154	.015	[.010, .020]	51.2%	1,499,736	85	.008	[.004, .012]	-15.4%	273,753	91
Employment in Agriculture	.016	[.013, .020]	100.0%	1,557,071	154	.013	[.009, .018]	72.3%	1,497,666	83	.014	[.008, .020]	-38.9%	271,963	90
Urban Population	.017	[.014, .021]	44.3%	1,557,071	154	.015	[.010, .019]	53.4%	1,499,377	84	.014	[.008, .021]	-88.4%	271,963	90

Table S3. Indirect effect of main-text model 4 when including covariates. Presented are estimates for the mediation effect of national religiosity when including various covariates, 95% Confidence Intervals, percentage mediated, final number of participants.

	GWP				IPP			
	Positive Emotions		Negative Emotions		Anxiety		Depression	
	beta	**95% CI**	***beta***	**95% CI**	***beta***	**95% CI**	***beta***	**95% CI**
Intercept	-.024	[-.061, .014]	-.117	[-.148, -.086]	-.211	[-.237, -.184]	-.109	[-.135, -.084]
SES	.174	[.164, .183]	-.204	[-.214, -.194]	-.072	[-.079, -.066]	-.043	[-.049, -.038]
Religiosity	.046	[-.002, .093]	.085	[.046, .125]	-.008	[-.040, .024]	.027	[-.003, .058]
Log10 GDP per capita	.103	[.055, .151]	.020	[-.020, .059]	.014	[-.018, .046]	.022	[-.009, .052]
Age	-.109	[-.110, -.107]	.036	[.034, .037]	-.072	[-.073, -.071]	-.116	[-.117, -.115]
Gender	.018	[.015, .021]	.085	[.082, .088]	.388	[.386, .390]	.217	[.214, .219]
SES x Religiosity	-.018	[-.030, -.006]	.008	[-.005, .020]	.022	[.014, .030]	.014	[.008, .021]
SES x Log10 GDP per capita	.012	[-2e-04, .024]	-.034	[-.047, -.022]	-.005	[-.013, .004]	-.012	[-.020, -.004]

Table S4. Estimates and 95% Confidence Intervals for main text model 3 when using alternative well-being indicators in the GWP and IPP. The WVS did not include any additional indicators of well-being besides the one used in the main text results. The final sample sizes for the models including alternative well-being indicators are reported in Table S5.

Alternative Well-Being Indicators	*beta*	95% CI	% mediated	*N*	Nations
GWP: Positive Emotions	.009	[.007, .011]	41.8%	1,545,102	156
GWP: Negative Emotions	-.003	[-.005, -.001]	9.3%	1,526,879	156
IPP: Anxiety	-.015	[-.019, -.011]	100.0%	3,169,763	116
IPP: Depression	-.010	[-.013, -.007]	50.8%	3,169,576	116

Table S5. Estimates, 95% Confidence Intervals, and percentage mediated for the results of main text model 4 when using alternative well-being indicators in the GWP and IPP. The WVS did not include any additional indicators of well-being besides the one used in the main text results.

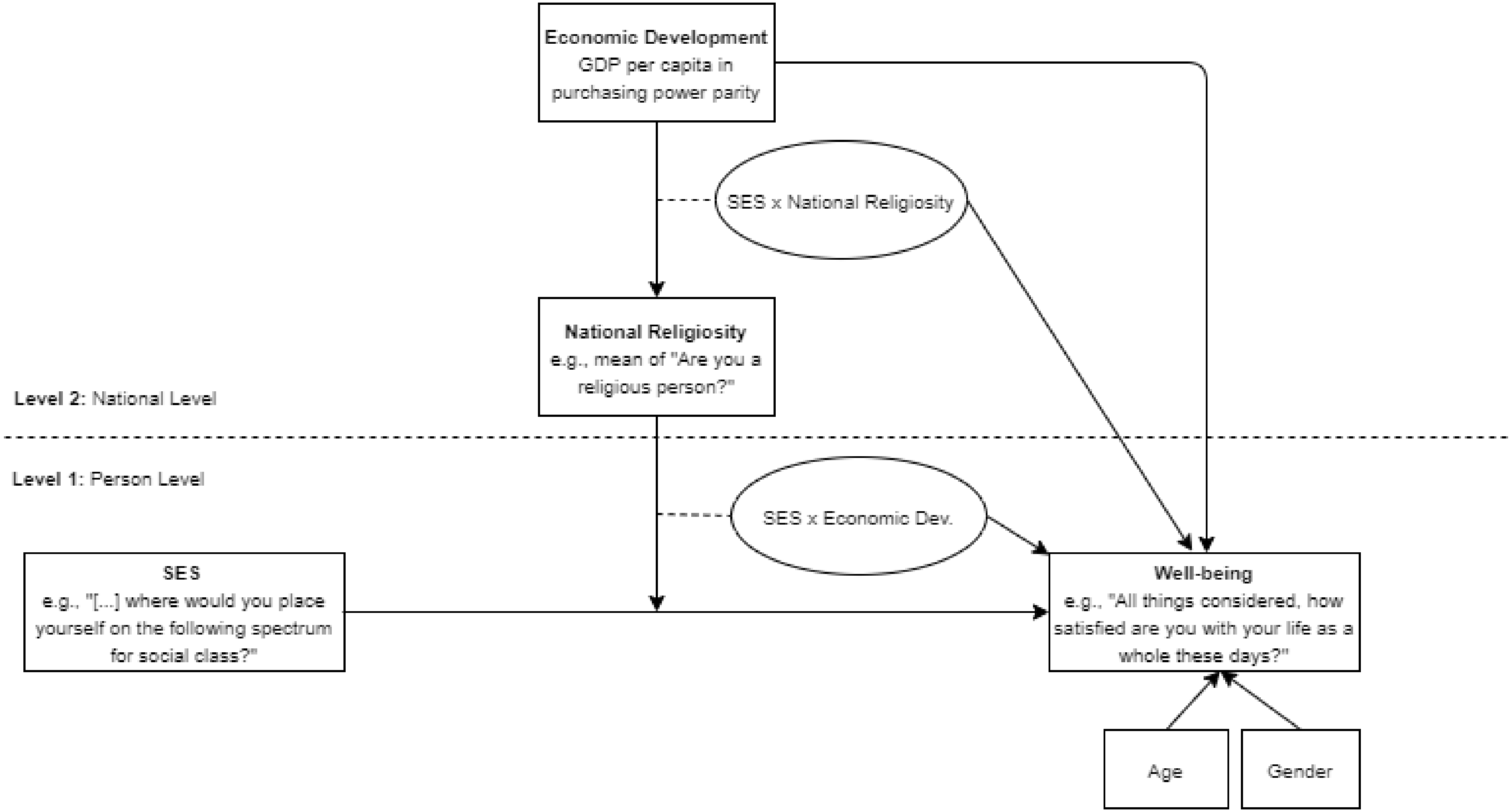

Figure S1. Mixed Effects Mediated Moderation Model (model 4). Statistical model to calculate the portion of the cross-level interaction (moderation) effect of economic development that is mediated by national religiosity.

SI References

1. T. M. Entringer, *et al.*, Big Five facets and religiosity: Three large-scale, cross-cultural, theory-driven, and process-attentive tests. *J. Pers. Soc. Psychol.* (2020) https:/doi.org/10.1037/pspp0000364.

2. J. E. Gebauer, *et al.*, The well-being benefits of person-culture match are contingent on basic personality traits. *Psychol. Sci.* **31**, 1283–1293 (2020).
